# MicroRNA-27a targets Sfrp1 to induce renal fibrosis in diabetic nephropathy by activating Wnt/β-Catenin signalling

**DOI:** 10.1042/BSR20192794

**Published:** 2020-06-15

**Authors:** MingJun Shi, PingPing Tian, ZhongQiang Liu, Fan Zhang, YingYing Zhang, LingLing Qu, XingMei Liu, YuanYuan Wang, XingCheng Zhou, Ying Xiao, Bing Guo

**Affiliations:** 1 State Key Laboratory of Functions and Applications of Medicinal Plants, Guizhou Medical University, Guiyang, Guizhou 550025, China; 2 Department of Pathophysiology, Guizhou Medical University, Guiyang, Guizhou 550025, China; 3 Laboratory of Pathogenesis Research, Drug Prevention and Treatment of Major Diseases, Guizhou Medical University, Guiyang, Guizhou 550025, China; 4 Guizhou Provincial People’s Hospital, Guiyang, Guizhou 550025, China

**Keywords:** diabetic nephropathy, miRNA-27a, renal fibrosis, Sfrp1, Wnt/β-catenin signaling pathway

## Abstract

Diabetic nephropathy (DN) commonly causes end-stage renal disease (ESRD). Increasing evidence indicates that abnormal miRNA expression is tightly associated with chronic kidney disease (CKD). This work aimed to investigate whether miR-27a can promote the occurrence of renal fibrosis in DN by suppressing the expression of secreted frizzled-related protein 1 (Sfrp1) to activate Wnt/β-catenin signalling. Therefore, we assessed the expression levels of miR-27a, Sfrp1, Wnt signalling components, and extracellular matrix (ECM)-related molecules *in vitro* and *in vivo*. Sfrp1 was significantly down-regulated in a high-glucose environment, while miR-27a levels were markedly increased. A luciferase reporter assay confirmed that miR-27a down-regulated Sfrp1 by binding to the 3′ untranslated region directly. Further, NRK-52E cells under high-glucose conditions underwent transfection with miR-27a mimic or the corresponding negative control, miR-27a inhibitor or the corresponding negative control, si-Sfrp1, or combined miR-27a inhibitor and si-Sfrp1. Immunoblotting and immunofluorescence were performed to assess the relative expression levels of Wnt/β-catenin signalling and ECM components. The mRNA levels of Sfrp1, miR-27a, and ECM-related molecules were also detected by quantitative real-time PCR (qPCR). We found that miR-27a inhibitor inactivated Wnt/β-catenin signalling and reduced ECM deposition. Conversely, Wnt/β-catenin signalling was activated, while ECM deposition was increased after transfection with si-Sfrp1. Interestingly, miR-27a inhibitor attenuated the effects of si-Sfrp1. We concluded that miR-27a down-regulated Sfrp1 and activated Wnt/β-catenin signalling to promote renal fibrosis.

## Introduction

The latest statistics of the International Diabetes Federation show that approximately 425 million people around the world had diabetes in 2017, and 629 million diabetes patients are projected by 2045 [1]. With increasing incidence rates of obesity and diabetes mellitus (DM), diabetic nephropathy (DN) is becoming more common on a global scale. DN, one of the prominent microvascular complications of DM, represents the main causative factor of end-stage renal disease (ESRD), which severely affects patients’ quality of life. The pathogenesis of DN remains unclear; therefore, further assessing DN pathogenesis and developing effective treatment measures are urgently needed.

The Wnt signalling pathway plays an important role in biological development and is involved in the processes of cell apoptosis, migration, invasion and differentiation [2,3]. Wnt signalling regulation has been extensively studied and described. In the normal physiological state, this pathway is activated and suppressed in an orderly manner. In addition to the canonical Wnt/β-catenin signalling pathway, non-canonical planar cell polarity and Wnt/Ca^2+^ pathways have been reported. In Wnt/β-catenin signalling, in the absence of the Wnt signalling factor, the β-catenin protein in the cytoplasm is inhibited by the degradation complex. This complex is made up of axin, adenomatous polyposis coli protein (APC), casein kinase 1 (CK1) and glycogen synthase kinase 3 (GSK3) β. CK1 and GSK3β carry out β-catenin phosphorylation and inactivation, causing β-catenin degradation by β-transducin repeat-containing protein (β-Trcp) and keeping β-catenin at a low level in the cytoplasm and out of the nucleus. When Wnt/β-catenin signalling is induced, the active Wnt binds to the membrane, which triggers the binding of its frizzled receptor (Frz) and the co-receptor LDL-receptor-related protein 5/6 (LRP5/6). Frz can act on dishevelled protein (Dsh/Dvl), leading to β-catenin dissociation from the degradation complex, cytosolic accumulation and translocation into the nucleus, where it activates downstream targets such as fibrosis-associated molecules, including fibronectin (FN), matrix metalloproteinase-7 (MMP-7), Twist, and Snail. Abnormal changes in the Wnt signalling pathway can cause many kidney diseases such as renal carcinoma, acute kidney injury, and renal fibrosis [4–6]. In the process of kidney injury, multiple factors cause epithelial cells to transform into fibroblasts or myofibroblasts, resulting in a loss of renal tubules and ECM deposition. Therefore, inhibiting or reversing ECM accumulation has become a major tool for controlling renal tubular interstitial fibrosis.

The secreted Frizzled-related protein (Sfrp) family is composed of secreted glycoproteins showing structural homology to the cysteine-rich domain (CRD) of Frz in Wnt signalling; therefore, they can compete with the latter for Wnt binding. The binding of Wnt ligands to Sfrps inhibits the canonical and non-canonical Wnt signalling pathways [7]. Sfrps control cell proliferation and differentiation and show low expression levels in a variety of tumour tissues [8–10]. Therefore, Sfrps are considered to form a class of tumour suppressors. Sfrp1, a member of the Sfrps family, is considered the most important target gene of miR-27a [11,12].

MicroRNAs (miRNAs) are highly conserved and short (approximately 19–25 bp) non-coding RNAs that specifically recognize target sequences in the 3′ untranslated regions (UTRs) of mRNAs, initiate mRNA degradation and cause post-transcriptional gene silencing [13–16]. MiRNAs regulate multiple genes and participate in several critical biological processes [17–20], including cellular proliferation, migration, invasion, apoptosis, and differentiation. They contribute to the occurrence and progression of chronic kidney diseases (CKDs) [18,19,21–23]. MiR-27a has been newly identified as an important factor in kidney disease [22]. However, the mechanism by which miR-27a regulates Wnt/β-catenin signalling to cause renal fibrosis remains largely unknown. Therefore, the present work aimed to assess the expression levels of miR-27a, Sfrp1, and Wnt/β-catenin signalling-related molecules *in vitro* and *in vivo* to investigate whether miR-27a can cause renal fibrosis by regulating Sfrp1 and activating Wnt/β-catenin signalling in high-glucose-treated renal tubular epithelial cells. Our findings provide new insights and an experimental basis for elucidating the pathogenesis of renal fibrosis.

## Materials and methods

### Animals

Twelve healthy and clean male Sprague-Dawley rats (180 ± 20 g) were obtained from LiaoNing Changsheng Biotechnology Co., Ltd. (LiaoNing, China) and approved by the Ethics Committee of Guizhou Medical University (Guizhou, China). The present study followed the guidelines of the National Health and Medical Research Council’s Code of Practice for the Care and Use of Animal Science. After 1 week of adaptive feeding in Guizhou Medical University, the 12 animals were randomized into the DN (*n*=6) and negative control (NC) (*n*=6) groups. The DN model was established as described previously [24]. Before modelling, the rats were weighed and fasted for 6–8 h. Under ether anaesthesia, streptozotocin (STZ; Sigma, U.S.A.) was administered by tail-vein injection in the DN group at 55 mg/kg. In the NC group, the vehicle (equivalent volume) was injected via the tail vein. After 72 h, tail-vein blood was collected, and blood glucose ≥16.7 mmol/l reflected successful establishment of the diabetic rat model. Qualitative examination of urine protein was performed 1 week later, and positive urine protein was considered to indicate renal damage, meaning that the DN rat model was successfully established. Each group was provided standard feed and free access to water. After 10 weeks, 24-h urine samples were collected before killing, Urine volumes were recorded, and 24-h urinary albumin were quantified. After fasting for 6–8 h, rats were anaesthetized with ether and then killed by bloodletting through the femoral artery. At the same time, femoral artery blood was collected to measure biochemical parameters. The kidneys were washed with normal saline until they appeared pale and weighed. The kidney index (KI) is the ratio of kidney weight to body weight. One kidney was fixed with 4% paraformaldehyde solution. The other was stored at −80°C for further analysis.

### Biochemical assays

The glucose oxidase method was used for the determination of serum blood glucose levels. Urine albumin (UAlb) was measured by pyrogallol colorimetry. The product of UAlb and 24-h urine volume was 24 h UAlb.

### Histology

Kidney tissues were fixed with 4% formaldehyde, dehydrated with an alcohol gradient, treated with xylene, paraffin embedded and sectioned at 3 µm. The morphological changes in kidney tissues were observed under a light microscope after haematoxylin–eosin (H&E) and Masson staining.

### Cell culture and transfections

Rat renal tubular epithelial cells (NRK-52E, American Type Culture Collection, Rockville, MD) were maintained in Dulbecco’s modified Eagle’s medium (DMEM; Gibco, U.S.A.) supplemented with 10% foetal bovine serum (Gibco, U.S.A.) in a humid atmosphere with 5% CO_2_ at 37°C. When the cells grew to 70% confluency, they were divided into the normal glucose (NG, 5.5 mM glucose), high glucose (HG, 30 mM glucose), miR-27a mimic (miR-27a m), miR-27a mimic negative control (miR-27a mnc), miR-27a inhibitor (miR-27a i), miR-27a inhibitor negative control (miR-27a inc), si-sfrp1, and miR-27a inhibitor/si-sfrp1 co-transfection (miR-27a i+si-sfrp1) groups. All groups were transfected for 4–6 h, and the medium was refreshed; protein and RNA samples were prepared after 48 h. The miR-27a mimic and inhibitor and their respective negative controls were provided by Guangzhou RiboBio Co., Ltd. (Guangdong, China). si-Sfrp1 was made by GenePharma (Shanghai, China). Lipofectamine™ 2000 (Invitrogen, U.S.A.) was used as directed by the manufacturer.

### Western blot analysis

Total protein from the kidney tissue and NRK-52E cells was obtained with a protein extraction kit (Solarbio, Beijing, China) as directed by the manufacturer. Sodium dodecyl sulphate–polyacrylamide gel electrophoresis (SDS-PAGE) was performed to separate proteins (50 μg/well). When bromophenol blue ran to the bottom of the gel, proteins were transferred onto polyvinylidene fluoride (PVDF) membranes. Blocking was carried out with 5% skim milk. The membranes were then washed with TBST three times for 10 min and subjected to incubation with the following antibodies overnight: β-catenin (1:500, bs-1165R, Bioss), Sfrp1 (1:500, bs-1303R, Bioss), p-GSK3β^ser9^ (1:500, sc-373800, Santa Cruz), E-cadherin (1:500, bs-10009R, Bioss), and α-SMA (1:500, 55135-1-AP, Proteintech), collagen IV (col-IV) (1:1000, SAB4200500, Sigma), β-actin (1:4000, Lot:181620, Pumei), and GSK3β (1:500, sc-8257, Santa Cruz). The next day, the membranes underwent three TBST washes of 10 min. Secondary antibodies diluted with 1% skim milk were added to the membranes for 1 h at room temperature. Band intensities were assessed by Image Lab software.

### Quantitative real-time PCR (qPCR)

Total RNA extraction from renal tissue specimens and NRK-52E cells was carried out with TRIzol reagent (Invitrogen, U.S.A.). A RevertAid™ First Strand cDNA Synthesis Kit (Thermo, U.S.A.) was employed for cDNA production. Subsequently, Talent qPCR PreMix (SYBR Green) (Tiangen, Beijing, China) was employed for qPCR. The corresponding primer sequence is shown in Table 1. Reverse transcription and quantitative detection of miR-27a were performed based on the protocols included in the RevertAid™ First Strand cDNA Synthesis Kit and Bulge-Loop™ miRNA qRT-PCR Primer Kit (RiboBio, Guangzhou, China), respectively. Relative quantification was carried out with the 2^−ΔΔCt^ method; U6 or β-actin served as an internal control.

**Table 1:** The primer sequences for qPCR

Gene name	Primer Sequence	*T*_m_
β-Actin	F: GCCAACACAGTGCTGTCT	63.9°C
	R: AGGAGCAATGATCTTGATCTT	
Sfrp1	F: CTCTCCAAGTGGCAGAGGAC	63.9°C
	R: GAGGACAGTCGCTGGAGTTC	
E-cadherin	F: ACTTTGGTGTGGGTCTGGAG	60.3°C
	R: TCTGTGGCAATGATGAGAGC	
α-SMA	F: GGCATCCACGAAACCACCT	60.3°C
	R: CCGCCGATCCAGACAGAAT	
col-IV	F: AAGACCTTGGTACTCTGGGC	54.0°C
	R: AGTAATTGGGGCCATGTCCA	

### Immunohistochemistry and immunofluorescence

For immunohistochemistry, a two-step immunoassay kit (ZSBIO, Beijing, China) was used for streptavidin-peroxidase (SP) detection. For immunofluorescence, NRK-52E cells were grown in 12-well plates, stimulated with normal or high glucose, and transfected with miR-27a inhibitor, miR-27a inhibitor negative control, si-sfrp1, and combined miR-27a inhibitor and si-sfrp1. After treatment for 48 h, the cells underwent fixation (4% formalin at room temperature, 20 min), permeabilization (0.1-0.3% Triton X-100, 10 min), and blocking (3% BSA at 37°C, 30 min). Next, sfrp1 antibody (1:50; bs-1303R, Bioss), β-catenin antibody (1:50; bs-1165R, Bioss), E-cadherin antibody (1:50; bs-10009R, Bioss), α-SMA antibody (1:50; 55135-1-ap, Proteintech), and col-IV antibody (1:50; SAB4200500, Sigma) were added for overnight incubation at 4°C. The cells were then rinsed with ice-cold PBS, and the corresponding fluorescent secondary antibodies were added for incubation (37°C, 1 h). DAPI staining was performed for 5 min, and the cells were observed under an immunofluorescence microscope (Leica, DM4000B, Germany).

### Dual-luciferase reporter assay

The day before transfection, NRK-52E cells were seeded onto 24-well plates and randomized into four groups. Transfection was carried out with miR-27a mimic or miR-27a mimic negative control at a concentration of 50 nM with 20 ng of Renilla plasmid (Promega, Wisconsin, U.S.A.) and 200 ng of wild-type (wt) or mutant (mt) plasmid containing the 3′ UTR of Sfrp1 (Era Biotech, Shanghai, China). Following 48 h of incubation, luciferase assays were carried out according to the Dual-Luciferase® Reporter Assay System (Promega) manual. The ratio of firefly to Renilla luciferase activity was assessed.

### Statistical analysis

SPSS 17.0 and the GraphPad Prism 5 statistical software were employed for all statistical analyses. Assays were repeated at least three times. Data are mean ± SD. Differences between two groups were analysed by independent-samples *t* test, and multiple groups were compared by one-way analysis of variance. *P*<0.05 indicated statistical significance.

## Results

### Successful model replication and increased miR-27a expression and decreased Sfrp1 expression *in vitro* and *in vivo* under high glucose

**Figure 1:**
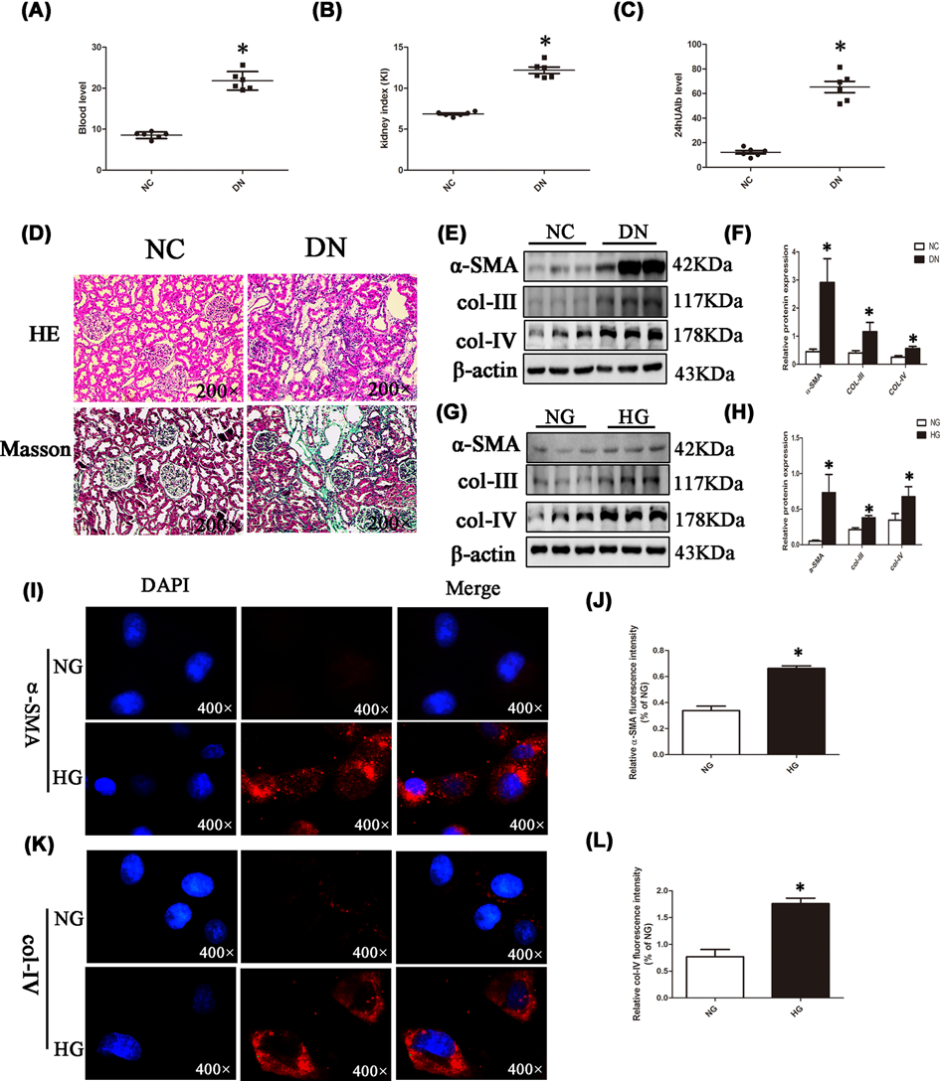
Evaluation of the extent of fibrotic lesions (**A–C**) The levels of blood glucose, KI, and 24 h urine in the NC group and the DN group. (**D**) Renal pathological changes in rats in the NC group and the DN group (magnification, 200×). (**E** and **F**) Protein levels of α-SMA, col-III, and col-IV in NC group and DN group rats were detected by Western blotting and analysed by Image Lab. (**G** and **H**) NRK-52E cells were treated with normal glucose (NG, 5.5 mM) or high glucose (HG, 30 mM) for 48 h, cell protein was obtained, and protein levels of α-SMA, col-III, and col-IV were detected by Western blotting and analysed by Image Lab. (**I–L**) NRK-52E cells were stimulated with normal or high glucose for 48 h, and then immunofluorescence of α-SMA and col-IV was detected and analysed by ImageJ (magnification, 400×); *P<0.05.

**Figure 2:**
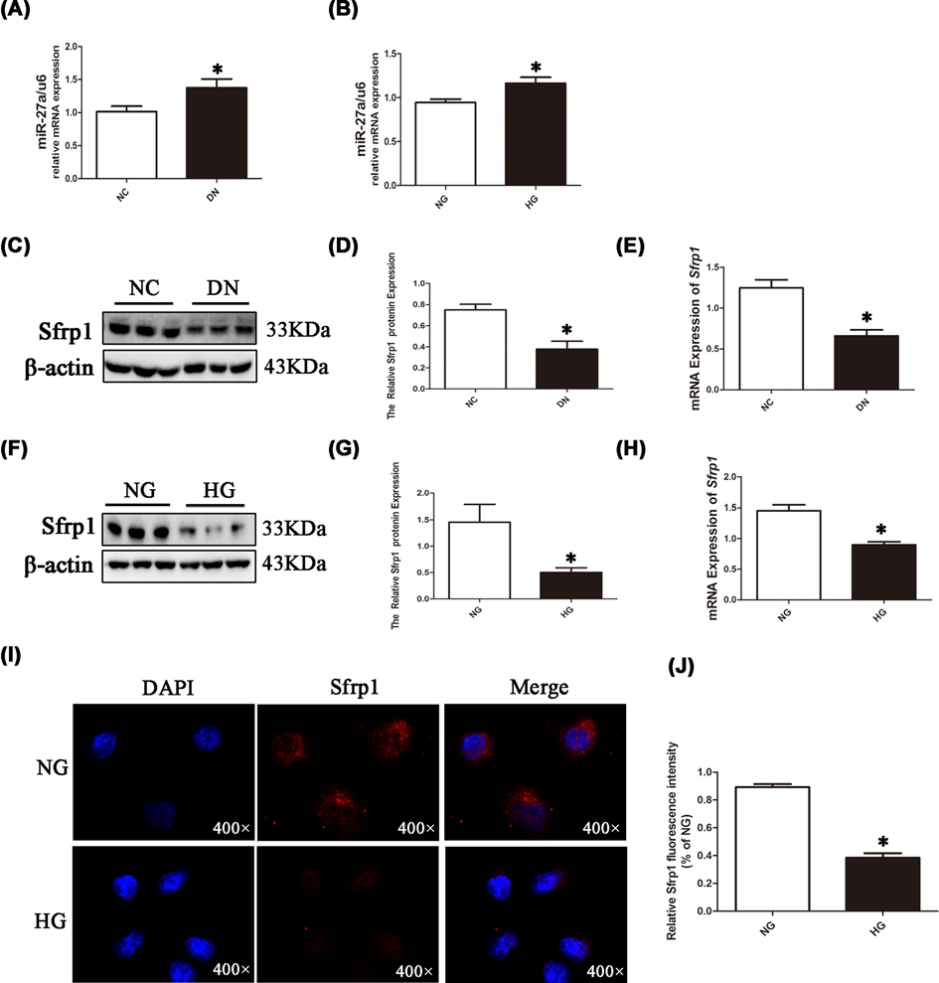
The expression of miR-27a and Sfrp1 in DN rats and NRK-52E cells (**A** and **B**) NRK-52E cells were treated with normal glucose (NG, 5.5 mM) or high glucose (HG, 30 mM) for 48 h, and RNA was extracted based on the protocol. The mRNA level of miR-27a was detected by qPCR *in vivo* and *in vitro.* (**C** and **D**) Total protein from the kidney tissue was obtained with a protein extraction kit. The protein expression level of Sfrp1 in NC group and DN group rats was detected by Western blotting and analysed by Image Lab. (**E**) Total RNA extraction from renal tissue specimens was carried out with TRIzol reagent. The RNA expression level of Sfrp1 in NC group and DN group rats was detected by qPCR. (**F** and **G**) NRK-52E cells were treated as described in Figure 2A,B, and cell protein was extracted based on the protocol. The protein level of Sfrp1 was analysed by Western blotting. (**H**) NRK-52E cells were treated as described in Figure 2A,B, and cell RNA was extracted as described in Figure 2E. The RNA expression level of Sfrp1 was detected by qPCR. (**I** and **J**) NRK-52E cells were stimulated with normal or high glucose for 48 h, and then immunofluorescence of Sfrp1 was detected and analysed by ImageJ (magnification, 400×); *P<0.05.

Three days following STZ administration, the rats had elevated blood glucose levels. In addition, compared with the NC group, the model rats showed other signs including increased drinking, food intake, and urine volume as well as weight loss. Meanwhile, blood glucose, KI, and 24 h urinary protein were elevated in the DN group in comparison with the NC group (Figure 1A–C). H&E staining showed that renal tubules in the NC group had a clear structure, with neatly arranged renal tubular epithelial cells and an intact basement membrane. In the DN group, some tubular epithelial cells exhibited vacuole degeneration and dilation; Masson staining demonstrated renal fibrosis in DN rats (Figure 1D). Moreover, immunoblotting showed that α-SMA, col-III, and col-IV protein levels were elevated in the DN group (Figure 1E,F). Similar results were obtained with NRK-52E cells (Figure 1G,H). The immunofluorescent signals of col-IV and α-SMA were obviously higher in the HG group in comparison with the NG group (Figure 1I–L). The above results suggested that the DN rat model and the NRK-52E cell model were successfully constructed. Therefore, we further performed Western blotting and/or qPCR to detect miR-27a and Sfrp1 levels. miR-27a levels were remarkably increased (Figure 2A,B) and Sfrp1 was significantly down-regulated in renal tissue samples from DN rats (Figure 2C–E) and high glucose-treated NRK-52E cells (Figure 2F–H) compared with the corresponding controls. The fluorescence intensity of Sfrp1 was weaker in the HG group in comparison with the NG group (Figure 2I,J).

### Wnt/β-catenin signalling pathway is active in a high-glucose environment

**Figure 3:**
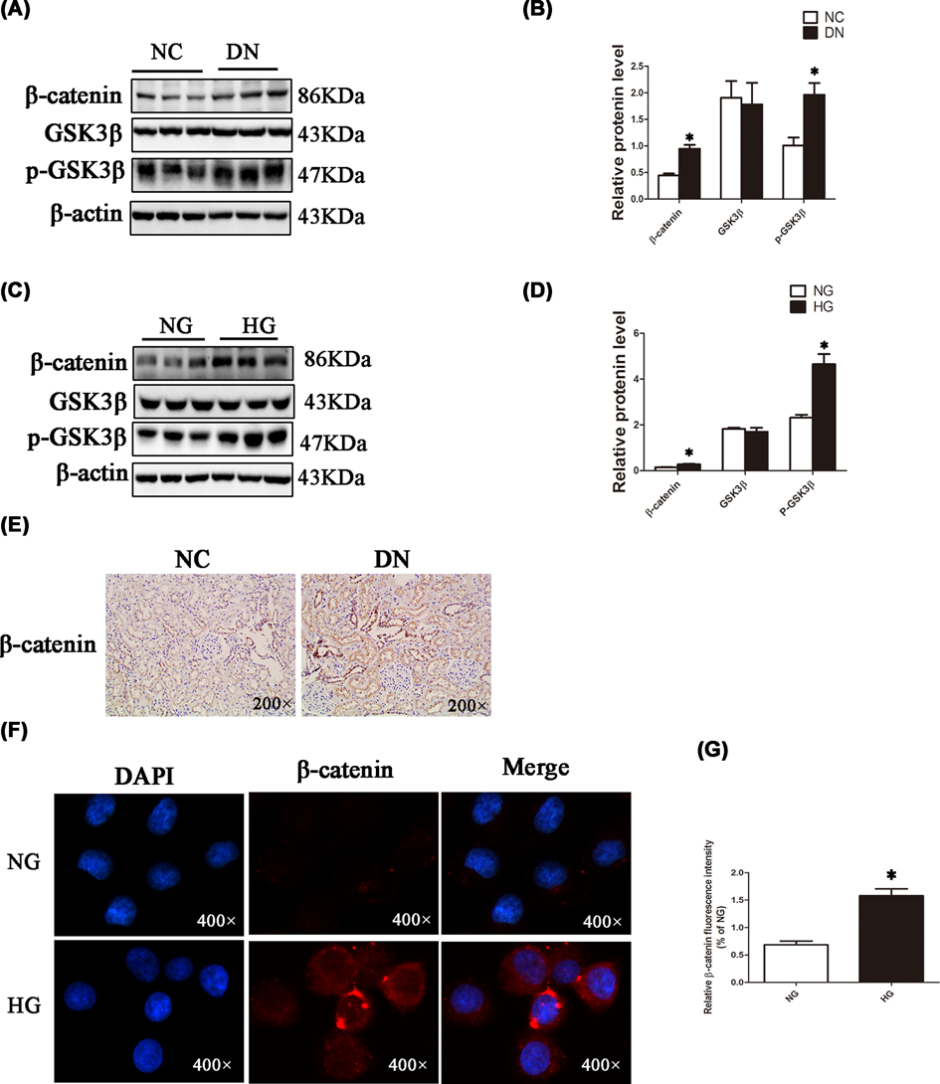
Expression of proteins in the Wnt/β-catenin signalling pathway in renal tissues and NRK-52E cells (**A** and **B**) The relative protein levels of the Wnt/β-catenin pathway were detected by Western blot analysis in NC group and DN group rats. (**C** and **D**) NRK-52E cells were stimulated with normal or high glucose for 48 h, cell protein was obtained, and relative protein levels of the Wnt/β-catenin pathway were detected by Western blotting and analysed by Image Lab. (**E**) Immunohistochemical analysis of β-catenin expression in renal tissues in the NC group and DN group (magnification, 200×). (**F** and **G**) NRK-52E cells were stimulated with normal or high glucose for 48 h, and then immunofluorescence staining of β-catenin was detected (magnification, 400×); *P<0.05.

Immunoblotting revealed that p-GSK3β and β-catenin protein levels were overtly higher in the DN group in comparison with the NC group, but total GSK3β levels were comparable in both groups (Figure 3A,B). Similar results were obtained in NRK-52E cells (Figure 3C,D). Immunohistochemistry confirmed that the β-catenin protein was markedly up-regulated in the DN group in comparison with the NC group and showed clear nuclear translocation (Figure 3E). In addition, immunofluorescence showed that β-catenin was remarkably up-regulated in the HG group in comparison with the NG group, also with clear nuclear translocation (Figure 3F,G). The above findings suggested Wnt/β-catenin signalling is activated in NRK-52E cells cultured under high glucose and in the kidneys of DN rats.

### miR-27a targets Sfrp1

**Figure 4:**
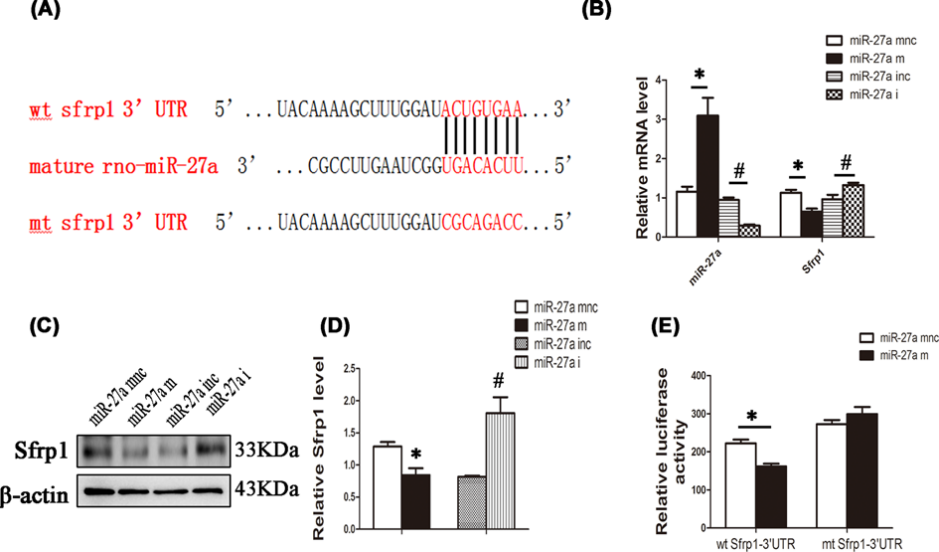
miR-27a targets Sfrp1 (**A**) miR-27a and its putative binding sequence in the 3′ UTR of Sfrp1. (**B**) NRK-52E cells were transfected with miR-27a mimic (miR-27a m), miR-27a mimic negative control (miR-27a mnc), miR-27a inhibitor (miR-27a i), or miR-27a inhibitor negative control (miR-27a inc). All groups were transfected for 4–6 h, the medium was refreshed, and RNA was extracted. The mRNA levels of miR-27a and Sfrp1 were detected by qPCR. (**C** and **D**) NRK-52E cells were transfected as described in Figure 4B, and protein was obtained. The protein expression level of Sfrp1 in different groups was detected by Western blotting and analysed by Image Lab. (**E**) NRK-52E cells were transfected with miR-27a mimic (miR-27a m) or miR-27a mimic negative control (miR-27a mnc) at a concentration of 50 nM with 20 ng of Renilla plasmid and 200 ng of wild-type (wt) or mutant (mt) plasmid containing the 3′ UTR of Sfrp1. A dual-luciferase reporter assay was performed to examine whether miR-27a targets Sfrp1 after 48 h; *P<0.05, ^#^*P*<0.05.

TargetScan and miRanda bioinformatic software were employed to predict the association of miR-27a with Sfrp1. We found that Sfrp1 mRNA may be a target of miR-27a (Figure 4A). First, miR-27a inhibitor and mimic, as well as the corresponding negative controls, were transfected into high glucose-treated NRK-52E cells. Then, immunoblotting and qPCR analyses were carried out to detect Sfrp1, and qPCR was performed for miR-27a quantitation. The results showed that miR-27a mimic reduced Sfrp1 RNA and protein levels. Conversely, miR-27a inhibitor reversed this phenomenon (Figure 4B–D). Luciferase reporter assays further confirmed miR-27a bound to the 3′ UTR of Sfrp1 and inhibited Sfrp1 expression directly (Figure 4E).

### miR-27a promotes the occurrence of renal fibrosis by targeting Sfrp1

**Figure 5:**
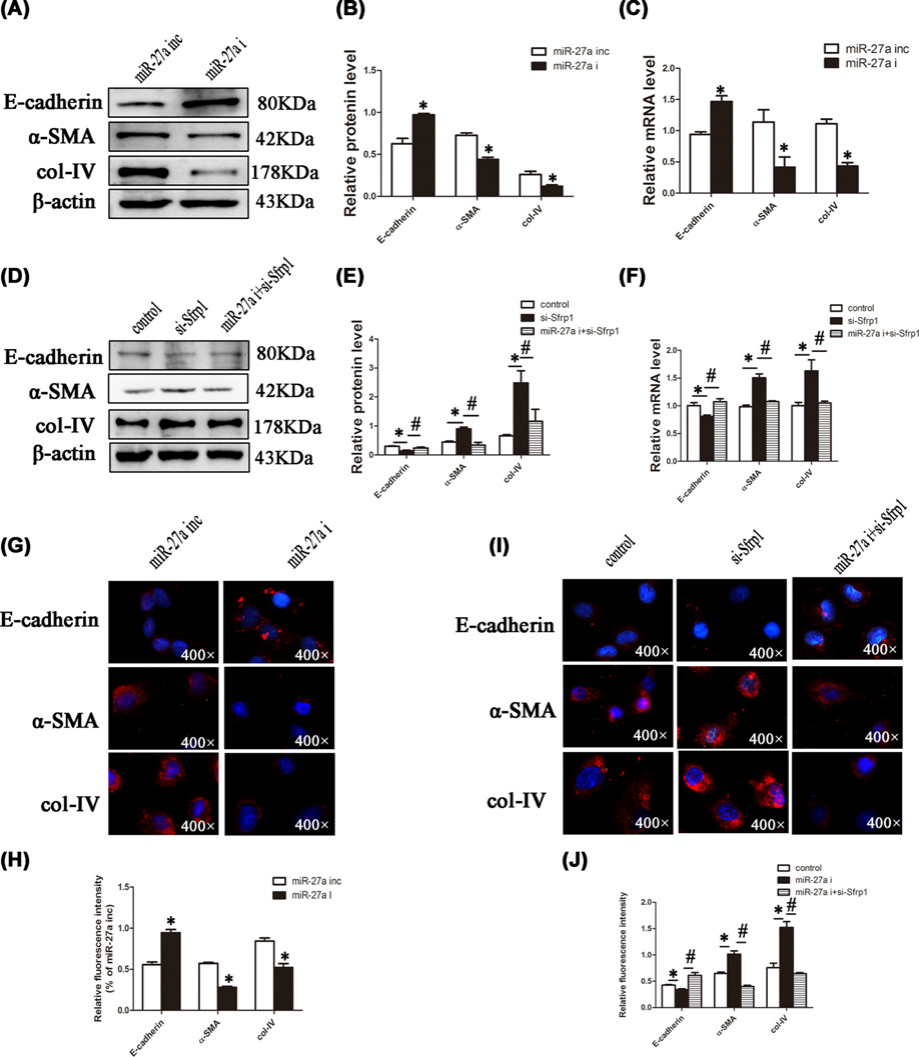
miR-27a can promote the occurrence of renal fibrosis by targeting Sfrp1 in NRK-52E cells (**A** and **B**) NRK-52E cells were transfected with miR-27a inhibitor (miR-27a i) or miR-27a inhibitor negative control (miR-27a inc). All groups were transfected for 4–6 h, and the medium was refreshed; protein was obtained after 48 h. The protein levels of fibrosis-related molecules in NRK-52E cells were detected by Western blotting and analysed by Image Lab. (**C**) NRK-52E cells were treated as described in Figure 4A, and RNA was extracted. The mRNA levels of fibrosis-related molecules were detected by qPCR. (**D** and **E**) NRK-52E cells were transfected with si-sfrp1 negative control, si-sfrp1, and miR-27a inhibitor/si-sfrp1 co-transfection (miR-27a i+si-sfrp1). The other methods are the same as described in Figure 4A, and the protein levels of fibrosis-related molecules were detected by Western blotting and analysed by Image Lab. (**F**) NRK-52E cells were treated as described in Figure 4D, and RNA was extracted. The mRNA levels of fibrosis-related molecules were detected by qPCR. (**G–J**) NRK-52E cells were transfected with miR-27a inhibitor (miR-27a i), miR-27a inhibitor negative control (miR-27a inc), si-sfrp1 negative control, si-sfrp1, and miR-27a inhibitor/si-sfrp1 co-transfection (miR-27a i+si-sfrp1). Immunofluorescence of E-cadherin, α-SMA, and col-IV was detected and analysed by ImageJ (magnification, 400×); *P<0.05, ^#^*P*<0.05.

Accordingly, we examined the expression levels of renal fibrosis-related molecules in NRK-52E cells. As shown in Figure 5A–C, miR-27a inhibitor decreased col-IV and α-SMA levels but increased E-cadherin levels. Conversely, si-Sfrp1 promoted the development of renal fibrosis, which was alleviated in the si-Sfrp1+miR-27a inhibitor co-transfection group (Figure 5D–F). Similar results were obtained by immunofluorescence (Figure 5G–J). The above findings suggested that miR-27a promoted the development of renal fibrosis through Sfrp1 repression.

### miR-27a targets Sfrp1 to promote renal fibrosis in DN by activating Wnt/β-catenin signalling

**Figure 6:**
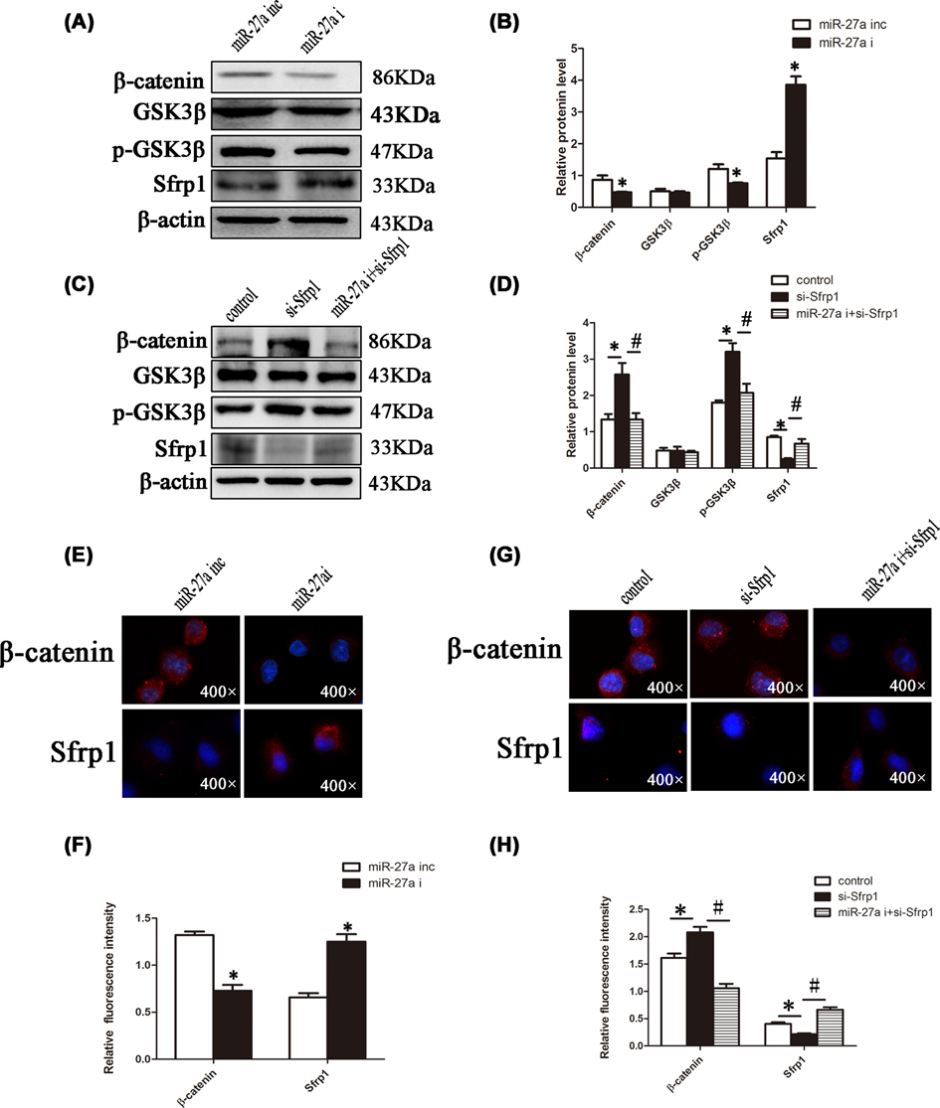
Downregulation of miR-27a inhibits the Wnt/β-catenin signalling pathway by upregulating Sfrp1 (**A** and **B**) NRK-52E cells were treated as described in Figure 5A, and protein was extracted. The protein levels of Wnt/β-catenin pathway-related molecules were detected by Western blotting and analysed by Image Lab. (**C** and **D**) NRK-52E cells were treated as described in Figure 5D, and protein was extracted. The protein levels of Wnt/β-catenin pathway-related molecules were detected by Western blotting and analysed by Image Lab. (**E–H**) NRK-52E cells were transfected as described in Figure 5G,J. Immunofluorescence of β-catenin and Sfrp1 was detected and analysed by ImageJ (magnification, 400×); *P<0.05, ^#^*P*<0.05.

To explore the specific mechanism by which miR-27a inhibits Sfrp1 and promotes renal fibrosis. miR-27a inhibitor, si-sfrp1, combined miR-27a inhibitor/si-sfrp1, and their respective negative controls underwent transfection into NRK-52E cells under high-glucose conditions, and the expression levels of Wnt/β-catenin signalling effectors were detected in each group. Western blot analysis showed that in the miR-27a i group, p-GSK3β and β-catenin levels were decreased (Figure 6A,B). Conversely, si-Sfrp1 up-regulated β-catenin and p-GSK3β, but this was markedly attenuated in the si-Sfrp1+miR-27a i group (Figure 6C,D). Similar results were obtained by immunofluorescence (Figure 6E–H). These results suggested that miR-27a induced Wnt/β-catenin signalling by down-regulating Sfrp1 and thus promoted renal fibrosis.

## Discussion

DN represents a progressive renal pathology due to the disturbance of glucose homeostasis that leads to structural and functional changes in glomerular capillaries and renal tubules. Although significant progress has been made in the treatment of diabetic nephropathy in recent decades, the pathogenesis remains unclear. Therefore, DN pathogenesis and efficient treatment are important topics for in-depth research.

The Wnt signalling pathway plays an important role in biological development. It contributes to the processes of cell apoptosis, migration, invasion, and differentiation in embryonic and tissue development [2,3]. The Wnt/β-catenin signalling pathway is closely related to the occurrence and development of fibrotic diseases [25–27]. The study found that the Wnt/β-catenin signalling pathway contributes to the activation of rat hepatic stellate cells and the occurrence of liver fibrosis [28]. Lu found that in TGF-β induced MSCs, the Wnt signalling pathway is activated and affects MSC differentiation to myofibroblasts [29]. He and co-workers [30] also found that the Wnt antagonist DKK1 reduces β-catenin deposition in UUO mouse kidneys as well as fibrosis occurrence. Ge [31] found that upon activation of HSCs, the expression of β-catenin is increased, and β-catenin siRNA inhibits the activation of HSCs and reduces the expression of col-I and col-III, which confirmed that the β-catenin plays an indispensable role in the development of liver fibrosis. β-Catenin is a key molecule of the Wnt/β-catenin signalling pathway that translocates into the nucleus and activates downstream target genes, eventually causing the occurrence of disease. This phenomenon is consistent with our experimental results.

Sfrps are secretory glycoproteins that regulate the Wnt signalling pathway. Studies have confirmed that Sfrps are involved in the regulation of cell proliferation and differentiation, showing low expression in a variety of tumour tissues [14,15]. Therefore, Sfrps are considered tumour suppressors. It also has been reported that the expression of the Wnt suppressor, Sfrp1, is down-regulated in colorectal cancer tissues, leading to the abnormal activation of the Wnt signalling pathway [32]. Mounting evidence suggests that Sfrp1 hypermethylation is a major mechanism for the down-regulation of Sfrp1 in cancer tissue [33–35]. It has also been confirmed that Sfrp1 is regulated via transcriptional silencing by microRNAs [36–39].

MiRNAs have recently attracted extensive attention. These molecules are involved in many essential life processes such as early development, cell invasion, apoptosis, lipid metabolism, and cellular differentiation [38–41]. MiRNAs cause mRNA degradation by binding to the 3′ UTR and then participate in the occurrence of multiple pathologies. They also have critical functions in renal fibrosis. In high glucose-treated glomerular mesangial cells and diabetic rat kidney tissues, Wu et al. found that miR-27 promotes ECM deposition through negative regulation at the 3′ UTR of PPAR-γ, and miR-27 antisense oligonucleotides could significantly reduce ECM deposition and proteinuria [22]. Meanwhile, after miR-27a inhibitor was transfected into glomerular mesangial cells, cell proliferation was remarkably reduced, suggesting miR-27a promotes the proliferation of mesangial cells and ECM deposition. Some studies suggest that Sfrp1 is the most important target gene of miR-27a [11,12]. MiR-27a down-regulates Sfrp1 and induces Wnt/β-catenin signalling, causing glioma [12]. Qiao and co-workers also demonstrated miR-27a-3p promotes epithelial–mesenchymal transition (EMT) occurrence by targeting Sfrp1 and activating the Wnt/β-catenin signalling pathway in oral squamous cell carcinoma [11]. In human colorectal cancer cells, miR-27a promotes cell proliferation and migration by regulating Sfrp1 to activate the Wnt/β-catenin signalling pathway [39]. However, the associations of miR-27a, Sfrp1, Wnt/β-catenin signalling, and renal fibrosis remain undefined. Therefore, DN rat and cell models were used to assess the functions of miR-27a in renal fibrosis, exploring the underlying mechanisms. TargetScan and miRanda bioinformatics software were employed to demonstrate that Sfrp1 might be a miR-27a target gene. Dual-luciferase reporter assays further revealed that miR-27a down-regulated Sfrp1 by binding to its 3′ UTR. Finally, high glucose-treated NRK-52E cells were transfected with si-sfrp1 negative control, si-sfrp1, and miR-27a inhibitor/si-sfrp1 co-transfection. The results showed that transfection with si-Sfrp1 induced Wnt/β-catenin signalling pathway, increased ECM deposition, and promoted the development of renal fibrosis, whereas co-transfection with miR-27a inhibitor attenuated these effects.

In conclusion, we found that miR-27a activates Wnt/β-catenin signalling by down-regulating Sfrp1, thus promoting the occurrence of renal fibrosis. This finding provides a new biomarker and a new therapeutic target for the treatment of DN.

## Abbreviations

CKD: chronic kidney diseases
DN: diabetic nephropathy
Dsh/Dvl: dishevelled
ECM: extracellular matrix
EMT: epithelial–mesenchymal transition
ESRD: end-stage renal disease
FN: fibronectin
GSK3β: glycogen synthase kinase 3β
LRP5/6: LDL-receptor-related protein5/6
miRNA: microRNA
MMP-7: matrix metalloproteinase-7
Sfrp1: secreted frizzled-related protein one
Sfrps: secreted frizzled-related protein family
